# *GNAQ/GNA11* Mosaicism Causes Aberrant Calcium Signaling Susceptible to Targeted Therapeutics

**DOI:** 10.1016/j.jid.2023.08.028

**Published:** 2024-04

**Authors:** Davide Zecchin, Nicole Knöpfel, Anna K. Gluck, Mark Stevenson, Aimie Sauvadet, Satyamaanasa Polubothu, Sara Barberan-Martin, Fanourios Michailidis, Dale Bryant, Asuka Inoue, Kate E. Lines, Fadil M. Hannan, Robert K. Semple, Rajesh V. Thakker, Veronica A. Kinsler

**Affiliations:** 1 Mosaicism and Precision Medicine Laboratory, Francis Crick Institute, London, United Kingdom; 2 Genetics and Genomic Medicine, UCL GOS Institute of Child Health, London, United Kingdom; 3 Department of Paediatric Dermatology, Great Ormond St Hospital for Children, London, United Kingdom; 4 Academic Endocrine Unit, Radcliffe Department of Medicine, University of Oxford, Oxford, United Kingdom; 5 Graduate School of Pharmaceutical Sciences, Tohoku University, Sendai, Japan; 6 Nuffield Department of Women's & Reproductive Health, University of Oxford, Oxford, United Kingdom; 7 Centre for Cardiovascular Science, Queen’s Medical Research Institute, University of Edinburgh, Edinburgh, United Kingdom; 8 National Institute for Health Research Oxford Biomedical Research Centre, Oxford, United Kingdom

## Abstract

Mosaic variants in genes *GNAQ* or *GNA11* lead to a spectrum of vascular and pigmentary diseases including Sturge-Weber syndrome, in which progressive postnatal neurological deterioration led us to seek biologically targeted therapeutics. Using two cellular models, we find that disease-causing *GNAQ*/*11* variants hyperactivate constitutive and G-protein coupled receptor ligand–induced intracellular calcium signaling in endothelial cells. We go on to show that the aberrant ligand-activated intracellular calcium signal is fueled by extracellular calcium influx through calcium-release-activated channels. Treatment with targeted small interfering RNAs designed to silence the variant allele preferentially corrects both the constitutive and ligand-activated calcium signaling, whereas treatment with a calcium-release-activated channel inhibitor rescues the ligand-activated signal. This work identifies hyperactivated calcium signaling as the primary biological abnormality in *GNAQ/11* mosaicism and paves the way for clinical trials with genetic or small molecule therapies.

## Introduction

Mosaic disorders are grouped by a common pathogenetic mechanism, namely a single-cell variant occurring during embryonic or fetal development which leads to a disease phenotype in a percentage of the body only (reviewed in [Kinsler et al, 2020]). Despite their monogenic nature, the wide variability in timing and cell lineage of the variant leads to protean clinical presentations, and gene discovery in the last decade has led to reclassification of many diagnoses into disease spectra (Keppler-Noreuil et al, 2015; Thomas et al, 2016).

*GNAQ* and *GNA11* mosaicism fits perfectly into this complex mould. We now understand that this disease spectrum causes a range of vascular and/or pigmentary abnormalities, affecting most commonly any combination of the skin, brain, and eyes. At the purely vascular end, diagnoses include Sturge-Weber syndrome (SWS) (Kalischer, 1901, Sturge, 1879, Weber, 1922), which if accompanied by specific pigmentary abnormalities is correctly named phakomatosis pigmentovascularis (subtypes associated with dermal melanocytosis [Happle, 2005; Hasegawa, 1979; Ota, 1947] or PPV-DM). A purely pigmentary phenotype also exists (Thomas et al, 2016), and both this and PPV-DM additionally carry an increased risk of melanoma. Over the last decade, SWS and PPV-DM have been discovered to be caused in most cases by heterozygous postzygotic mosaic variants in genes *GNAQ* or *GNA11* (Jordan et al, 2020; Polubothu et al, 2020; Shirley et al, 2013; Thomas et al, 2016). For unknown reasons *GNAQ* variants predominate in SWS, whereas *GNAQ* and *GNA11* are more evenly represented in PPV-DM (Jordan et al, 2020; Polubothu et al, 2020; Shirley et al, 2013; Thomas et al, 2016). Variants usually affect codon 183 of each gene, very rarely codon 209 (Galeffi et al, 2022; Thomas et al, 2016). *GNAQ* variants enriched in SWS endothelial cells (Huang et al, 2017) suggested that these were the cell of origin for the vascular end of the spectrum and supported the clinical observation that the vascular phenotype reflects embryonic vascular patterning (Waelchli et al, 2014).

This work focuses on the vascular phenotype in this spectrum. The neurovascular abnormalities in SWS and PPV-DM present with seizures, neurodevelopmental impairment, headaches, and stroke-like episodes (Comi, 2007). Importantly, symptoms often worsen during the first year of life, thought to be related to seizure-related damage as well as cerebral perfusion defects (Comi, 2007). This postnatal progression suggests a tantalising window at which to target therapy. Despite the genetic insights, there has been relatively little exploration of the downstream biology. In vitro studies in human embryonic kidney cells have demonstrated basal activation of MAPK signaling downstream of disease-causing *GNAQ/11* variants (Shirley et al, 2013; Thomas et al, 2016); however, this has not been confirmed in human endothelial cells (Fjær et al, 2021; Huang et al, 2022). The only animal modeling to date—in zebrafish—was restricted to recapitulating the pigmentary and not the vascular phenotype (Thomas et al, 2016).

We hypothesized that whatever the original mechanism leading to the congenital vascular malformation, postnatal neurological disease progression in SWS or PPV-DM may be associated with disturbed local calcium handling in variant endothelial cells. This hypothesis was based on several observations. Firstly, patients progressively develop neurovascular calcification, visualised as classical “tram-lining” of blood vessels on plain skull radiography (Weber, 1922). Secondly, the proteins encoded by *GNAQ* and *GNA11*, G subunit-aq and -a11 respectively, are known regulators of intracellular calcium signaling in other contexts. Lastly, pathogenic germline variants in *GNA11* cause familial hypocalciuric hypercalcaemia type 2 and autosomal dominant hypocalcaemia type 2 (Nesbit et al, 2013; Wettschureck et al, 2007). By modeling the commonest causative *GNAQ/GNA11* variants in endothelial cells, this work identifies calcium signaling as a fundamental downstream cellular abnormality. Design and testing of targeted genetic therapies and repurposing of a small-molecule therapy identifies this pathway as druggable, paving the way for clinical trials.

## Results

### *GNAQ/GNA11* variants cause constitutive activation of intracellular calcium signaling in endothelial cells

**Figure 1:**
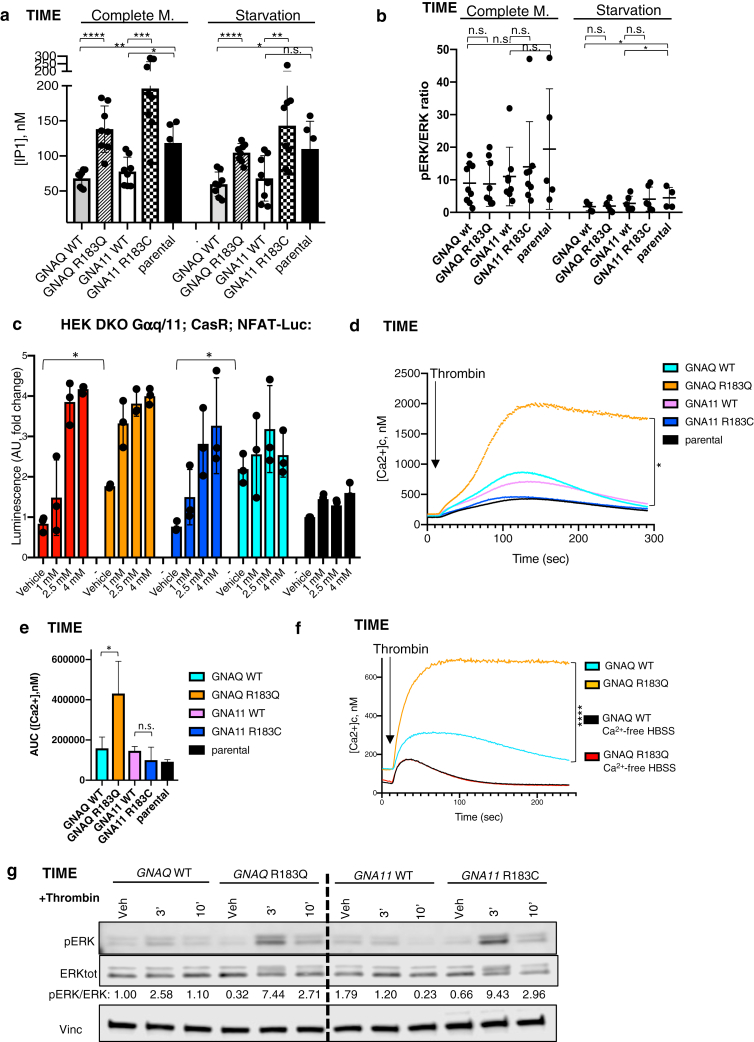
**Effect of *GNAQ/GNA11* variants on constitutive and ligand-induced intracellular calcium and ERK signaling in endothelial cells.** (**a**) TIME recombinant or parental cell lines were assayed for concentration of IP1 in complete medium and starvation conditions. The graph represents the mean ± SD of 8 independent experiments. Statistical comparison among conditions was by two-tailed unpaired *t*-test (∗∗∗∗*P* < .0001, ∗∗∗*P* = .0003, ∗∗*GNA11*^*WT*^ versus *GNA11*^*R183C*^*P* = .0059, ∗∗*GNA11*^*WT*^ versus parental *P* = .0025, ∗*P* < .05, n.s. = nonsignificant). (**b**) Densitometric analysis performed on a minimum of five independent western blot experiments on TIME cell lines in complete medium or after 1-hour acute starvation. Results are shown as mean ± SD. Two-tailed unpaired *t*-tests did not reveal statistically significant differences between *GNAQ or GNA11* WT and variant cell lines in any condition (n.s. = nonsignificant, ∗*P GNAQ*^*WT*^ versus parental starvation = .048, ∗*P GNA11*^*WT*^ versus parental starvation = .032). **(c)** HEK DKO Gaq/11; CaSR;NFAT-Luc cells were transfected with the *GNAQ*^*WT*^, *GNAQ*^*R183Q*^, *GNA11*^*WT*^, or *GNA11*^*R183C*^ constructs and treated with vehicle or three concentrations of extracellular calcium to stimulate activation of CaSR and downstream G-protein signaling. Luciferase activity was measured 4 hours after stimulation. The graph represents the mean ± SD of three independent experiments. Statistical comparison among different conditions was performed by two-tailed paired *t*-test (∗ *P* < .05). (**d**) TIME recombinant or parental cell lines were loaded with intracellular calcium probe Fluo-8 and stimulated with thrombin (1U/Ml) in HBSS standard buffer. Changes in fluorescence over the time were recorded and normalised to maximum and minimum responses to calculate cytosolic (Ca^2+^). The graph represents an average of three independent experiments performed with four technical replicates. Statistical test comparing *GNAQ*^*R183Q*^ and *GNAQ*^*WT*^ is described in (e). (**e**) Means ± SD deviation of areas under the curve calculated from three experiments summarised in Figure 1d. Statistical comparisons were performed by two-tailed unpaired *t*-test (n.s. = statistically nonsignificant, ∗ *P* = .049). (**f**) TIME-GNAQ^*WT*^ or *GNAQ*^*R183Q*^ were loaded with intracellular calcium probe Fluo-8 and stimulated with thrombin (1U/Ml) in HBSS standard buffer (yellow and blue lines) or after 100-second-long exposure to HBSS calcium-free buffer (black and red lines). Changes in fluorescence over the time were recorded and normalised to maximum and minimum responses to calculate cytosolic Ca^2+^ level. The graph represents an average of three independent experiments performed with six technical replicates. Statistical test performed by two-way ANOVA (∗∗∗∗*P* <.0001). (**g**) Western blot time-course analysis of TIME recombinant cell lines starved for 1 hour and treated by vehicle or thrombin (1U/Ml) for the times indicated. Lysates were probed with the indicated antibodies. Densitometric quantification of pERK/ERK bands showed increased activation of the pathway in both *GNAQ* and *GNA11* variant cells compared with WT counterparts following 3′ or 10′ treatment by thrombin. One representative of three independent experiments is shown. AUC, area under curve; ERK, extracellular signal–regulated kinase; HBSS, Hanks' Balanced Salt Solution; HEK, human embryonic kidney; IP1, inositol-1-phosphate; n.s., nonsignificant; pERK, phosphorylated ERK; TIME, telomerase-immortalised microvascular endothelial; WT, wild type.

**Supplementary Figure S1:**
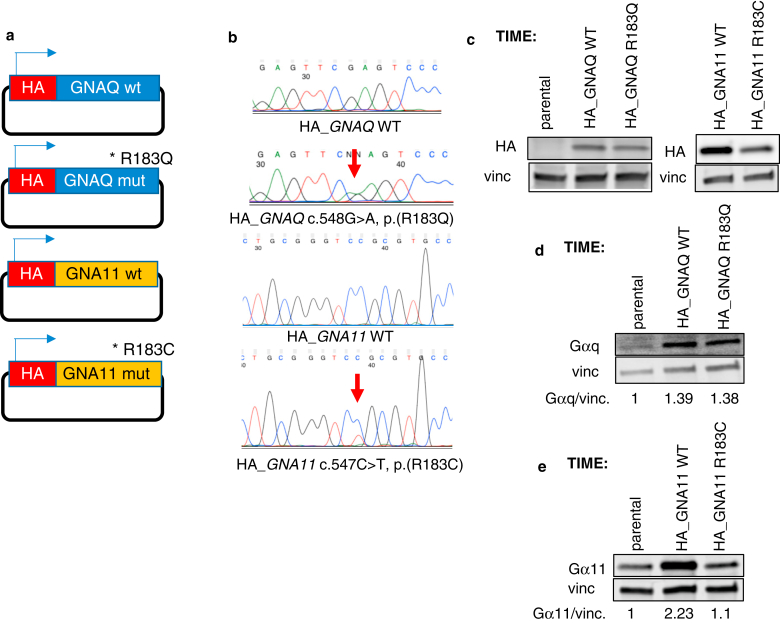
**Generation and validation of TIME transgenic cell lines stably expressing GNAQ^WT^, GNAQ^R183Q^, GNA11^WT^, or GNA11^R183C^ alleles.** (**a**) Schematic representation of the lentiviral expression vectors used to infect the TIME cell line and generate stable recombinant derivatives. (**b**) Sanger sequencing performed on TIME recombinant models using primers annealing to exon 4 of *GNAQ* or *GNA11* genes. Chromatogram of *GNAQ* and *GNA11* codon-183 for mutation confirmation. (**c**) Western blot analysis of TIME parental cells and TIME transduced with *GNAQ*^*WT*^, *GNAQ*^*R183Q*^, *GNA11*^*WT*^, or *GNA11*^*R183C*^ lentiviruses. The cell lysates were probed with the indicated antibodies. (**d**) Western blot analysis of TIME parental cells and TIME transduced with *GNAQ*^*WT*^ or *GNAQ*^*R183Q*^ lentiviruses. The cell lysates were probed with the indicated antibodies. Quantification of the bands reported in figure represents the average of four independent western blot experiments. (**e**) Western blot analysis of TIME parental cells and TIME transduced with *GNA11*^*WT*^ or *GNA11*^*R183C*^ lentiviruses. The cell lysates were probed with the indicated antibodies. Quantification of the bands reported in figure represents the average of four independent western blot experiments. ERK, extracellular signal–regulated kinase; HA, hemagglutinin; HEK, human embryonic kidney; mut, mutated; pERK, phosphorylated ERK; TIME, telomerase-immortalised microvascular endothelial; WT, wild-type.

**Supplementary Figure S2:**
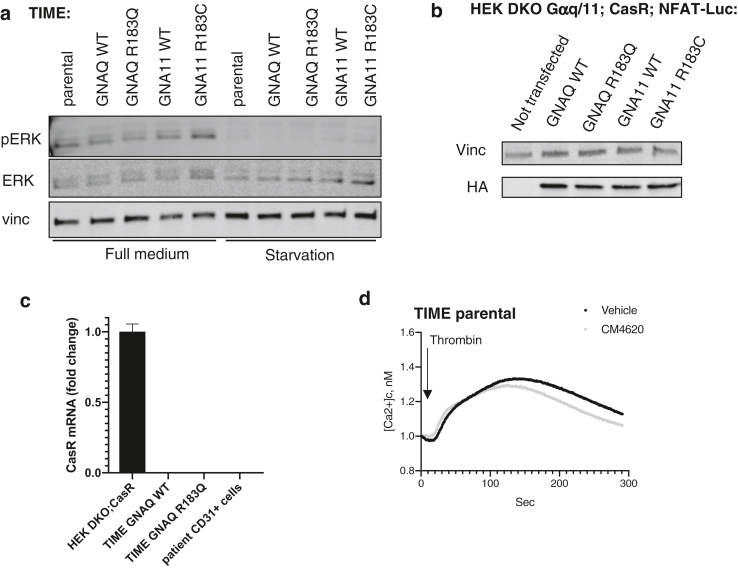
(**a**) One representative western blot among the ones considered in the densitometric analysis reported in Figure 1b. The cell lysates were probed with the indicated antibodies to show variations in pERK/ERK ratio. (**b**) Western blot analysis of HEK DKO G**α**q/11; CasR; NFAT-Luc cells nontransfected or transfected with GNAQWT, GNAQR183Q, GNA11^WT^, or *GNA11*^*R183C*^ pcDNA3.1(+)-N-HA plasmids. The cell lysates were probed with the indicated antibodies to show equal expression of the transgenes. (**c**) RT-qPCR analysis of CaSR expression levels in TIME *GNAQ* WT or *GNAQ* p.(R183Q) cell lines and in a CD31^+^ cell population isolated from a SWS vascular lesion. HEK DKO;CaSR were used as positive control of the TaqMan expression assay. Mean ± SD of technical quadruplicate of one experiment representative of two independent experiments. (**d**) TIME parental cells were loaded with intracellular calcium probe Fluo-8 and treated for 20 minutes with vehicle or 1 μM CM4620. After treatment, cells were stimulated with thrombin 1U/ml and fluorescence recorded for 300 seconds. The graphs represent an average of four independent experiments. ERK, extracellular signal–regulated kinase; HEK, human embryonic kidney; pERK, phosphorylated ERK; TIME, telomerase-immortalised microvascular endothelial; WT, wild-type.

Transgenic telomerase-immortalised microvascular endothelial (TIME) cells were used to characterise the effects of variants on calcium signaling. To this aim, TIME parental cells were transduced with lentiviral vectors to induce stable expression of hemagglutinin-tagged forms of *GNAQ* wildtype (WT), *GNAQ* p.(R183Q), *GNA11* WT, or *GNA11* p.(R183C) cDNAs (Supplementary Figure S1a and b). Transduced lines expressed WT/variant hemagglutinin-tagged transgenes at similar levels (Supplementary Figure S1c), and total expression of Gaq or Ga11 in the respective transgenic models was higher but within the same order of magnitude as endogenous expression observed in parental TIME cells (Supplementary Figure S1d and e). Strikingly, basal calcium signaling was highly significantly increased in both TIME-*GNAQ*^*R183Q*^ and TIME*-GNA11*^*R183C*^ variant cells compared to WT controls, as demonstrated by a sharp increase in inositol-1-phosphate accumulation in both complete and nutrient-deprived medium (Figure 1a). On the other hand, no differences were seen in basal MAPK activation between variant and WT (Figure 1b and Supplementary Figure S2a). Ectopic expression of *GNAQ* or *GNA11* WT transgenes decreased basal calcium signaling but had no effect on constitutive MAPK activation compared with TIME parental cells (Figure 1a and b).

To validate our findings in a second cellular system without interference from endogenous Gaq and Ga11, human embryonic kidney double knock out (DKO) Gaq/11;CaSR;nuclear factor of activated T-cells (NFAT)-Luc cells were transfected with vectors for expression of *GNAQ*^*WT*^, *GNAQ*^*R183Q*^, *GNA11*^*WT*^, or *GNA11*^*R183C*^ hemagglutinin-tagged cDNAs (Supplementary Figure S2b). This model, therefore, uses calcium as an extracellular G-protein coupled receptor (GPCR) ligand, which leads to intracellular calcium signaling. Untransfected cells were appropriately unresponsive to extracellular calcium stimulation, whereas transfected cells showed increased luciferase signal, validating the model (Figure 1c). Variant *GNAQ* and *GNA11* cells had significantly increased NFAT-driven luciferase signal compared with WT, in the absence of extracellular calcium (Figure 1c), confirming basal constitutive activation of calcium signaling.

Levels of *CASR* expression were undetectable in variant and WT *GNAQ* TIME cell lines, and in CD31^+^ cells isolated from a vascular cutaneous lesion of a patient with SWS (Supplementary Figure S2c).

### Variant *GNAQ* amplifies and prolongs GPCR-ligand–induced intracellular calcium signaling in endothelial cells, fueled by extracellular calcium influx

The dynamics of calcium signaling activation in TIME cells upon GPCR ligand stimulation were studied using thrombin as the prototypical GPCR stimulant in this cell type (Korhonen et al, 2009). TIME-*GNAQ*^*R183Q*^ showed significantly increased and prolonged levels of intracellular calcium compared with TIME-GNAQ^*WT*^ in response to thrombin stimulation, an effect not seen in TIME-*GNA11*^*R183C*^ (Figure 1d and e). Strikingly, this difference was entirely abolished by removing calcium from the extracellular buffer (Figure 1f), identifying influx of extracellular calcium as the reservoir for the ligand-induced aberrant signal. TIME-*GNAQ*^*R183Q*^ and -*GNA11*^*R183C*^ also showed increased levels of phosphorylated extracellular signal–regulated kinase or extracellular signal–regulated kinase after thrombin stimulation compared with their respective WT controls (Figure 1g)

### Variant allele–specific small interfering RNAs (siRNAs) rescue basal and ligand-induced aberrant calcium signaling in two endothelial cell models

**Figure 2:**
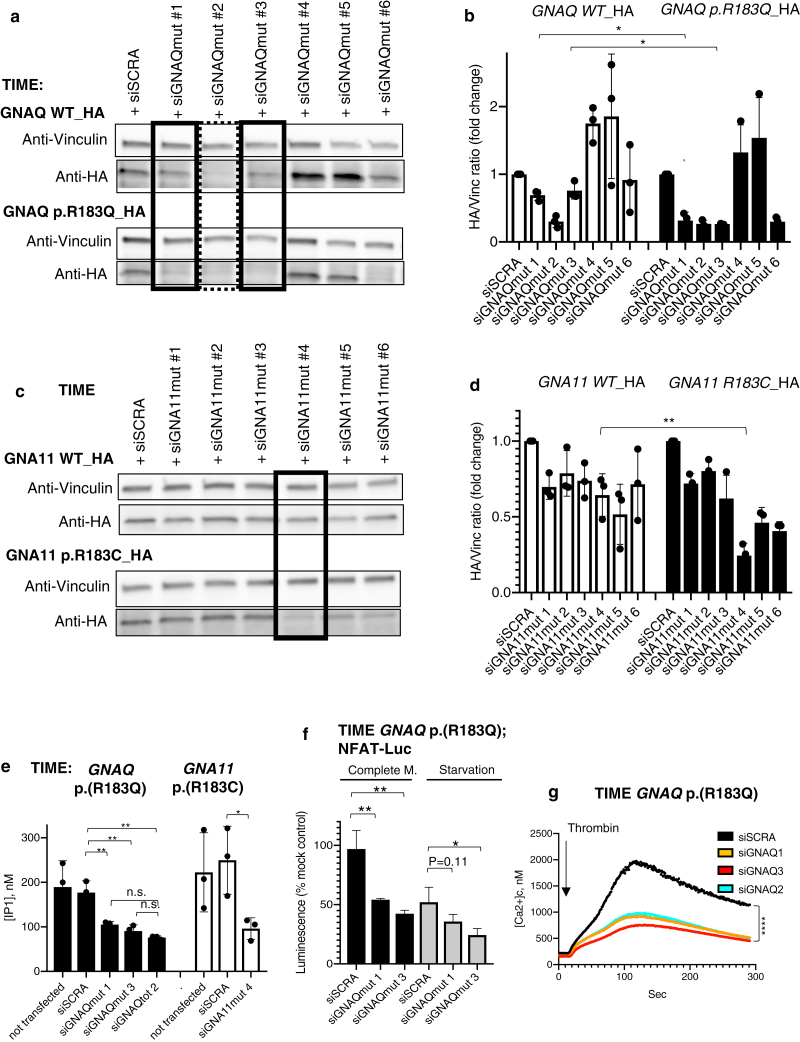
**Identification of siRNAs for specific targeting of *GNAQ* or *GNA11* variant alleles leads to rescue of aberrant calcium signaling in variant cells.** (**a**) TIME cells stably expressing either WT or p.(R183Q) HA-tagged Gaq were transfected by 50 nM siRNAs targeting *GNAQ* c.548G > A, p.(R183Q) allele and analysed by western blot 24 hours after transfection. Lysates were probed with the indicated antibodies. siRNAs siGNAQmut #1 and 3 (squared with solid lines) showed specific knockdown of variant protein over WT counterparts. siRNA siGNAQmut #2 (dotted square) knockeddown both variant and WT proteins. (**b**) Densitometric quantification of bands from western blot experiments similar to the ones shown in Figure 2a (mean ± SD of three experiments, ∗ *P* < .05). (**c**) TIME cells stably expressing either WT or p.(R183C) HA-tagged Ga11 were transfected by 25 nM siRNAs targeting *GNA11* c.547C > T, p.(R183C) allele and analysed by western blot 24 hours after transfection. Lysates were probed with the indicated antibodies. siRNAs siGNA11mut#4 (squared) showed specific knockdown of variant protein over WT counterparts. (**d**) Densitometric quantification of bands from western blot experiments similar to the ones shown in Figure 2c (mean ± SD of three experiments, ∗ *P* < .05). (**e**) TIME-*GNAQ*^*R183Q*^ or -*GNA11*^*R183C*^ were not transfected or transfected with 25 nM nontarget siRNAs (siSCRA), 25 nM siRNAs for specific silencing of the variant alleles (siGNAQmut 1 and siGNAQmut 3 for targeting of *GNAQ* variant allele and siGNA11mut 4 for silencing of *GNA11* variant allele), or 25 nM siRNA targeting both variant and WT *GNAQ* alleles (siGNAQ tot 2). IP1 concentration was measured 48-hours after transfection and shown as mean ± SD of three independent experiments. Statistical comparisons were performed by two-tailed unpaired *t*-test (∗∗*P* < .01, n.s.= nonsignificant). (**f**) TIME cells harbouring the *GNAQ*^*R183Q*^ variant were transfected with NFAT-luciferase reporter and a stable clone was obtained after antibiotic selection. TIME-*GNAQ* p.(R183Q); NFAT-Luc were transfected with nontarget siRNA (siSCRA) or two siRNAs for specific silencing of the variant *GNAQ* allele (siGNAQmut 1 and 3) and luciferase reporter activity was measured 48-hours after transfection in complete medium or after four hours of starvation, shown as mean ± SD of percentage change of cells transfected with mock in three independent experiments. Statistical comparisons were performed by two-tailed unpaired *t*-test (∗*P* < .05; ∗∗*P* < .01). (**g**) TIME cells harbouring *GNAQ*^*R183Q*^ were transfected with nontarget siRNA (siSCRA), two siRNAs for specific silencing of the variant *GNAQ* allele (siGNAQ1 and siGNAQ3), or siRNA targeting both variant and WT *GNAQ* alleles (siGNAQ2). Forty-eight hours after transfection they were loaded with Fluo-8 intracellular calcium dye and stimulated by thrombin 1U/ml while recording fluorescent signal at 1 second intervals for up to 300 seconds. The graph shows the average of three independent experiments. Statistical tests performed by one-way ANOVA (∗∗∗∗*P* < .0001). AUC, area under curve; ERK, extracellular signal–regulated kinase; HA, hemagglutinin; HEK, human embryonic kidney; IP1, inositol-1-phosphate; mut, mutated; n.s., nonsignificant; pERK, phosphorylated ERK; siRNA, small interfering RNA; siSCRA; nontarget siRNA; TIME, telomerase-immortalised microvascular endothelial; WT, wild type.

siRNAs were designed and optimised for specific knock-down of *GNAQ* c.548G>A, p.(R183Q) or *GNA11* c.547C>T, p.(R183C) transcripts while sparing the WT alleles, as a molecular tool to study the biological effects of these variants and as potential therapeutic agents for the treatment of these diseases. Two siRNAs which successfully knocked down variant Gaq protein and one targeting variant Ga11 over the respective WT counterparts were identified (Figure 2a–d). An siRNA targeting to the same extent both WT and variant *GNAQ* alleles was also identified (Figure 2a) and used as an additional control in further experiments. All three variant allele–specific siRNAs rescued constitutive basal calcium signaling activation in TIME *GNAQ*^*R183Q*^ and TIME *GNA11*^*R183C*^ cells as measured using the inositol-1-phosphate assay. The non-specific siRNA produced a similar effect as variant-specific siRNAs (Figure 2e), proving the prevalent effect of the variant protein on activation of the pathway.

To validate this result using a second assay, TIME-*GNAQ*^*R183Q*^ cells were engineered to incorporate stably a NFAT-luciferase calcium signaling reporter (Figure 2f), in which treatment with siRNA once again normalised basal calcium signaling. TIME-*GNAQ*^*R183Q*^ were then transfected with oligos and intracellular calcium accumulation measured after thrombin stimulation. The aberrantly-prolonged response of TIME-*GNAQ*–variant cells to thrombin was rescued to a similar extent by silencing of the variant transcript or by treatment with siRNA targeting both *GNAQ* alleles (Figure 2g), strongly tying the variant to both the basal and ligand-induced signaling abnormality.

### Calcium-release-activated channel (CRAC) inhibition rescues aberrant calcium signaling in variant cells

**Figure 3:**
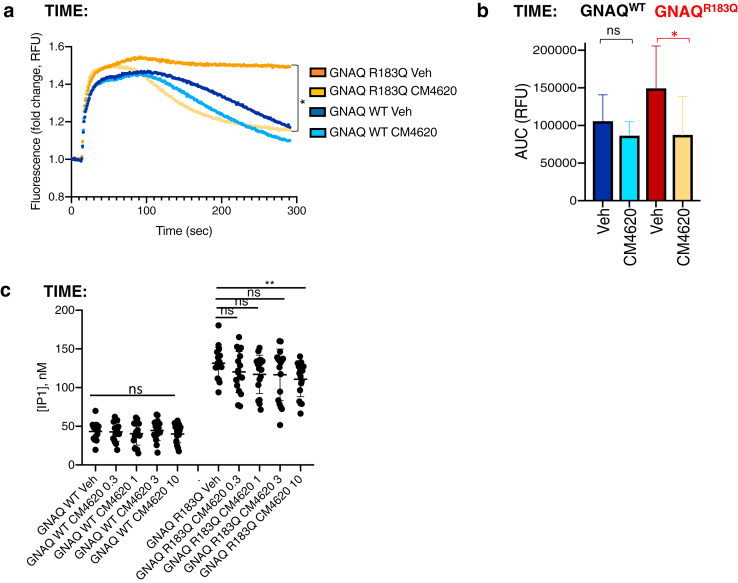
**CRAC inhibition rescues ligand-induced aberrant calcium signaling in variant cells.** (**a**) TIME-GNAQ^*WT*^ or *-GNAQ*^*R183Q*^ were loaded with intracellular calcium probe Fluo-8 and treated for 20 minutes with vehicle or 1 μM CM4620. After treatment, cells were stimulated with thrombin 1U/ml and fluorescence recorded for 300 seconds. The graphs represent an average of three independent experiments. Statistical test comparing *GNAQ*^*R183Q*^ treated by vehicle or CM4620 is described in (b). (**b**) Means ± SD of AUC calculated from three experiments summarised in Figure 3a. Statistical comparisons were performed by two-tailed paired *t*-test (n.s. = statistically nonsignificant, ∗*P* = .0284). (**c**) TIME recombinant cell lines were assayed for concentration of IP1 after treatment with vehicle or CM4620 0.3***μ***M, 1***μ***M, 3***μ***M, or 10***μ***M. The graph represents the mean ± SD of three independent experiments including 5–6 technical replicates each. Statistical comparison among conditions was performed by two-tailed unpaired *t*-test (n.s. = nonsignificant, ∗∗*P* = .0078). AUC, area under curve; IP1, inositol-1-phosphate; ns, nonsignificant; RFU, relative fluorescence unit; TIME, telomerase-immortalised microvascular endothelial; Veh, vehicle; WT, wild type.

Activation of G-proteins downstream of GPCRs in physiological conditions leads to generation of inositol tris-phosphate and opening of the intracellular inositol tris-phosphate–gated calcium channel of the endoplasmic reticulum (Michell, 1975; Michell et al, 1981). Endoplasmic reticulum emptying then triggers replenishment of calcium stores through opening of cell membrane CRAC and intracellular influx of extracellular calcium. We, therefore, hypothesized that increased activation of calcium signaling downstream of variant Gaq was driving influx of extracellular calcium through CRAC. In support of this, treatment with CRAC specific inhibitor Auxora (CM4260, CalciMedica, La Jolla, CA) markedly rescued the prolonged calcium intracellular peak in *GNAQ*-variant cells (Figure 3a and b), with only limited effects on thrombin-induced calcium signaling in TIME parental (Supplementary Figure S2d) or *GNAQ*^*WT*^ cells (Figure 3a and b). CRAC inhibition had comparatively little effect on basal calcium signaling and only at higher concentrations (Figure 3c).

### In vitro angiogenesis is disrupted by variant *GNAQ* and rescued by CRAC inhibition

**Figure 4:**
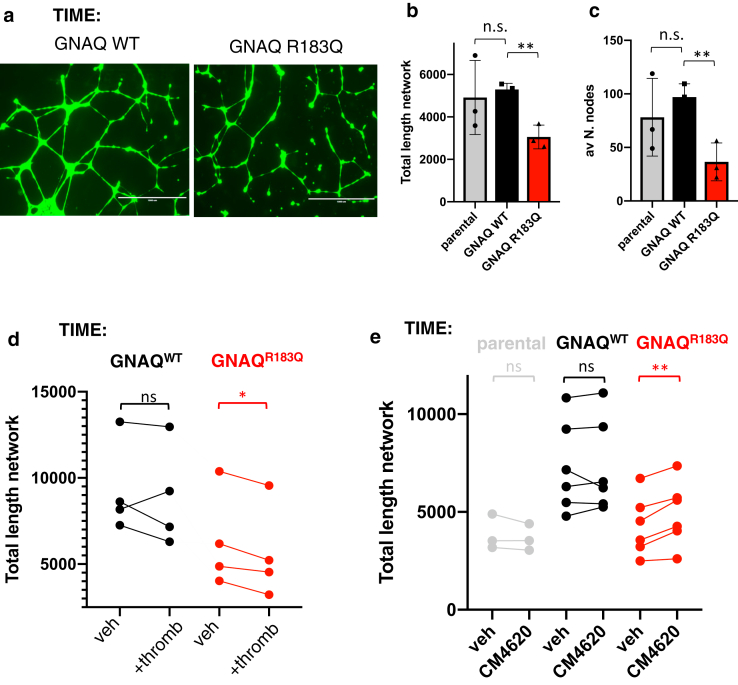
**In vitro angiogenesis is disrupted by variant *GNAQ* and improved by CRAC inhibition.** (**a**) Representative images captured with EVOS Floid Imaging System after Calcein AM staining during in vitro endothelial cell tube formation of TIME cells stably expressing either *GNAQ* WT or *GNAQ* R183Q. (**b** and **c**) Quantification of angiogenesis assays **(**average of three independent experiment, mean ± SD) demonstrates significant difference between WT and variant cells in total length of the network (defined as combined lengths of segments, branches, and isolated elements) (**b**) and in the average number of nodes in the network (**c**). Statistical significance calculated using two-tailed unpaired *t*-test on three independent experiments (∗∗*P* = .0035 in **b**, ∗∗*P* = .0083 in (**c**). (**d**) Quantification of angiogenesis assays (total length of the network, defined as combined lengths of segments, branches, and isolated elements, four experiments) demonstrates significant difference between vehicle and thrombin (0.3U/Ml)–treated TIME *GNAQ*^R183Q^, but no statistically significant difference for TIME *GNAQ*^WT^. Results shown as mean of a technical triplicate for each of 4 independent experiments. Statistical analysis was performed by two-tailed paired *t*-test on four independent experiments (n.s. = not significant, ∗*P* = .0125). (**e**) Quantification of angiogenesis assays (total length of the network, defined as combined lengths of segments, branches, and isolated elements, three experiments) performed in presence of thrombin 0.3U/Ml demonstrates significant difference between vehicle and CM4620 (1 mM)–treated TIME *GNAQ*^R183Q^, but no statistically significant difference for parental or *GNAQ*^WT^ TIME. Results shown as three or six independent experiments, and statistical analysis was performed by two-tailed paired *t*-test (n.s. = statistically nonsignificant, ∗∗*P* = .0048). n.s., nonsignificant; TIME, telomerase-immortalised microvascular endothelial; WT, wild type.

TIME endothelial cell models were then used to assess angiogenesis using a standard in vitro angiogenesis assay (Arnaoutova and Kleinman, 2010). TIME-*GNAQ*^*R183Q*^ cells had significantly impaired tubule formation in basement membrane matrix (Figure 4a–c), linking the variant not only to the pathogenesis of the vascular malformations but also to the postnatal cellular behaviour. Furthermore, thrombin-GPCR activation disrupted angiogenesis in TIME-*GNAQ*^R183Q^ more than in TIME-GNAQ^WT^ (Figure 4d). The impact of siRNA knock-down of the variant allele on angiogenic properties was technically very difficult to discern because of nonspecific toxicity of in vitro transfection and the impact on survival of variant cells challenged by new conditions. Treatment with CRAC inhibitor CM4620; however, significantly improved tubule formation specifically in TIME-*GNAQ*^R183Q^ (Figure 4e), though the effect size was modest.

## Discussion

Despite the congenital nature of the vascular malformations, the *GNAQ/11* mosaicism spectrum frequently has progressive postnatal neurological deterioration (Comi, 2007), suggestive of a secondary, dynamic, and ongoing disease process which might be preventable or treatable. This deterioration is often associated with progressive accumulation of neurovascular calcification that appears likely to contribute to the problem of chronic anoxia underlying the abnormal cerebral vasculature. Indeed, both levels of intracranial calcification and venous hypoperfusion on radiological studies have been correlated with neurological symptoms (Kelley et al, 2005; Lin et al, 2006; Pilli et al, 2017). This led us to hypothesize that brain calcifications may be signs of disturbed local calcium homeostasis, and that this process may contribute to the postnatal progression of the disease.

Our results clearly demonstrate that *GNAQ* disease variants induce marked constitutive and GPCR ligand–induced hyperactivation of intracellular calcium signaling in microvascular endothelial cells. We confirm previous negative results investigating the effect of variants on basal MAPK signaling (Fjær et al, 2021; Huang et al, 2022), but demonstrate GPCR ligand–induced activation of MAPK. The calcium signaling abnormalities extend across the plasma membrane of variant cells into the surrounding environment, inducing massively increased influx of calcium from the extracellular space through CRAC. The exact mechanism linking aberrant influx of calcium inside variant cells with accumulation of mineral deposits within the lesions is currently unknown and deserves further investigation, possibly by using animal models or organothropic coculture systems. Interestingly, the same variants lead also to disrupted angiogenesis in vitro, implicating abnormal calcium signaling in the pathogenesis of the vascular malformations as well as the postnatal phenotype.

Interestingly the results for TIME-*GNA11*^*R183C*^ differed in one regard from those of TIME-*GNAQ*^*R183Q*^. Basal calcium signaling, basal MAPK signaling, and ligand-induced MAPK signaling were the same as for *GNAQ*, however, thrombin-induced calcium signaling was not different between TIME-*GNA11*^*R183C*^ and TIME-*GNA11*^*WT*^ cells. This raises the interesting possibility that G subunit-aq and -a11 calcium signaling is triggered by different ligand-GPCR interactions, a hypothesis potentially supported by subtle differences in the vascular phenotype of patients with *GNAQ* and *GNA11* mosaicism (Jordan et al, 2020).

These findings led us on to design and testing of two therapeutic approaches to our knowledge previously unreported. In the first we designed and screened siRNAs using a tiling approach spanning the variants, and identified variant allele–specific siRNAs for both *GNAQ* and *GNA11* causative variants. As a highly-targeted therapy directed to the root cause of disease, these siRNAs rescued both the baseline and the ligand-induced calcium signaling abnormalities in vitro. Indeed, siRNAs molecules, unlike other approaches targeting downstream effectors, should be able to correct any potential downstream signaling effect of variant *GNAQ/GNA11.* siRNA therapies are increasingly appearing in clinical trials and clinical practice (Esrick et al, 2021; Sardh et al, 2019), and these previously undescribed molecules offer the prospect of a genetic therapy approach in this mosaic disorder.

In the second therapeutic approach, CRAC inhibitor Auxora (CM4260, CalciMedica) was used to block the influx of extracellular calcium, rescuing the aberrant intraendothelial calcium signaling in response to ligand stimulation in TIME-*GNAQ*^*R183Q*^. Blockage of CRAC may be a potential therapeutic approach and clinical trials are currently being explored, because CM4620 is already in phase 2 clinical trials for the treatment of pancreatitis-associated hypocalcaemia (trial NCT04195347). In our study, CRAC inhibitor was also effective in improving angiogenesis of variant endothelial cells. However, the rescue was only partial, suggesting that other effectors downstream variant G proteins, may affect the functional properties of endothelial cells.

Taken together these findings demonstrate clearly that the postnatal phenotype of vascular *GNAQ/GNA11* mosaic disorders are primarily diseases of calcium signaling and calcium handling across variant cellular membranes and that these pathways are druggable. The biological abnormalities could conceivably be involved in the classical chronic progressive neurocalcification and the postnatal clinical deterioration. These insights, therefore, offer potential therapeutic opportunities in postnatal disease progression in SWS and PPV-DM.

## Materials and Methods

### Cell lines

hTERT-immortalised microvascular endothelial cells (TIME-ATCC CRL-4025, “TIME”) and their transgenic derivatives were authenticated by short tandem repeat DNA profiling. TIME cell lines were maintained in EBM-2 Endothelial Cell Growth Basal Medium-2 (Lonza, Basel, Switzerland; CC-3156), supplemented with EGM-2 BulletKit (Lonza CC-3162), and 3% fetal bovine serum (Gibco, Carlsbad, CA).

TIME parental cells were transduced with lentiviral vectors to induce stable expression of hemagglutinin-tagged forms of *GNAQ* WT, *GNAQ* p.(R183Q), *GNA11* WT, and *GNA11* p.(R183C) cDNAs (Supplementary Figure S1a), confirmed by Sanger sequencing (Supplementary Figure S1b).

Human embryonic kidney DKO Gaq/11; CaSR;NFAT-Luc cells were derived as follows: human embryonic kidney DKO Gaq/11 lacking functional *GNAQ* and *GNA11* (Schrage et al, 2015) were engineered to integrate NFAT-luciferase calcium reporter stably and to overexpress the CaSR, and maintained in DMEM-Glutamax media (Thermo Fisher Scientific, Waltham, MA) with 10% fetal bovine serum (Gibco), 400 μg/ml Geneticin (Thermo Fisher Scientific) and 100ug/ml hygromycin (Thermo Fisher Scientific).

## Data availability statement

All data are available in the main text and supplementary materials.

## ORCIDs

Davide Zecchin: http://orcid.org/0000-0002-4784-0336

Nicole Knöpfel: http://orcid.org/0000-0002-6438-6550

Anna K. Gluck: http://orcid.org/0000-0003-3459-2764

Mark Stevenson: http://orcid.org/0000-0001-8616-0205

Aimie Sauvadet: http://orcid.org/0000-0002-8980-1239

Satyamaanasa Polubothu: http://orcid.org/0000-0001-7195-5670

Sara Barberan-Martin: http://orcid.org/0000-0003-0142-4078

Fanourios Michailidis: http://orcid.org/0000-0002-0408-3603

Dale Bryant: http://orcid.org/0000-0002-4783-4796

Asuka Inoue: http://orcid.org/0000-0003-0805-4049

Kate E. Lines: http://orcid.org/0000-0002-0764-8681

Fadil M. Hannan: http://orcid.org/0000-0002-2975-5170

Robert K. Semple: http://orcid.org/0000-0001-6539-3069

Rajesh V. Thakker: http://orcid.org/0000-0002-1438-3220

Veronica A. Kinsler: http://orcid.org/0000-0001-6256-327X

## Conflict of Interest

The authors state no conflict of interest.

## Supplementary Materials and Methods

*GNAQ/GNA11* mosaicism causes aberrant calcium signaling susceptible to targeted therapeutics.

### Plasmids and reagents

*GNAQ* wildtype (WT), *GNAQ* c.548G>A, p.(R183Q), *GNA11* WT, and *GNA11* c.547C>T, p.(R183C) cDNAs were synthesized and cloned into a pcDNA3.1+ N-hemagglutinin (HA) plasmid, fused in-frame at their N-terminus with an HA tag (Genscript, Piscataway, NJ). Luciferase open reading frame was excised from pLenti PGK V5-LUC Puro (Addgene, Watertown, MA; 21471) by SalI and XbaI combined restriction digestion, and HA-tagged *GNAQ/11* cDNAs were amplified and cloned into the digested pLenti-vector using the In-Fusion HD Cloning kit (Takara Bio, Shiga, Japan; cat. 638947), following the on-line primer design tool and the manufacturer's instructions. The following antibodies were used: anti-phospho-ERK T202/Y204 (cat. 9101, 1:1000) and anti-ERK (cat. 9107, 1:1000) from Cell Signaling Technology (Danvers, MA); anti-vinculin (cat. MA5-11690, 1:3000) from Invitrogen (Waltham, MA), anti-HA (clone 16B12, cat. 901501, 1:2000) from BioLegend (San Diego, CA), and anti-Gaq (cat. sc-136181, 1:200) from Santa Cruz Biotechnology (Dallas, TX). CM4620 was obtained from MedChemExpress (Monmouth Junction, NJ; cat. HY-101942).

### Lentiviral particles production and transduction

Lentiviral particles were produced by transfecting HEK293T cells in 10-cm tissue culture dishes with 0.93 mg pCMV-VSVG, 2.79 mg delta-8.2 (Addgene) and 3.72 mg pLenti *GNAQ*^*WT*^, *GNAQ*^*R183Q*^, *GNA11*^*WT*^ or *GNA11*^*R183C*^ mixture (Lipofectamine, Invitrogen). After 48 hours of transfection, virus particles in the supernatant were harvested and stored at −80 ºC. Telomerase-immortalised microvascular endothelial (TIME) cells were transduced with *GNAQ*^*WT*^, *GNAQ*^*R183Q*^, *GNA11*^*WT*^, or *GNA11*^*R183C*^ lentiviral particles in 6-well tissue culture dishes, in the presence of 8 mg/ml polybrene and then selected using 4 mg/Ml puromycin.

### Generation of transgenic TIME cell lines

TIME parental cells were transduced with lentiviral vectors to induce stable expression of HA-tagged forms of *GNAQ* WT, *GNAQ* p.(R183Q), *GNA11* WT, and *GNA11* p.(R183C) cDNAs (Supplementary Figure S1a), and presence of the mutations in the genomic DNA confirmed by Sanger sequencing (Supplementary Figure S1b). Transduced lines expressed WT or mutant forms of HA-tagged transgenes at similar levels, and total expression of *GNAQ*-encoded protein Gaq in both *GNAQ* transgenic models was close to endogenous expression observed in parental TIME cells (Supplementary Figure S1c–e).

### Design and testing of mutation-specific small interfering RNAs (siRNAs)

Six siRNAs specifically annealing to mutant *GNAQ* c.548G > A, p.(R183Q) transcript were synthesized with the following sense strand sequences:

siGNAQmut #1: UGCUUAGAGUUCAAGUCCC[dT][dT]; siGNAQmut #2: GCUUAGAGUUCAAGUCCCC[dT][dT]; siGNAQmut #3: CUUAGAGUUCAAGUCCCCA[dT][dT]; siGNAQmut #4: UUAGAGUUCAAGUCCCCAC[dT][dT]; siGNAQmut #5: UAGAGUUCAAGUCCCCACC[dT][dT]; siGNAQmut #6: AGAGUUCAAGUCCCCACCA[dT][dT].

Six siRNAs specifically annealing to mutant *GNA11* c.547C>T, p.(R183C) transcript were synthesized with the following sense strand sequences:

siGNA11mut #1: GUGCUGCGGGUCUGCGUGC[dT][dT]; siGNA11mut #2: UGCUGCGGGUCUGCGUGCC[dT][dT]; siGNA11mut #3: GCUGCGGGUCUGCGUGCCC[dT][dT]; siGNA11mut #4: CUGCGGGUCUGCGUGCCCA[dT][dT]; siGNA11mut #5: UGCGGGUCUGCGUGCCCAC[dT][dT]; siGNA11mut #6: CGGGUCUGCGUGCCCACCA[dT][dT].

TIME transgenic cells were transfected with siRNAs using Lipofectamine RNAiMAX (Invitrogen) following manufacturer’s instructions.

We assayed these panels of mutant-specific siRNAs for their ability to discriminate between WT and mutant transcripts of *GNAQ* or *GNA11* by transfecting TIME cells expressing the respective HA-tagged transgenes and measuring the knockdown of the WT or mutant HA-tagged proteins.

Two of six siRNAs designed to anneal specifically to the mutant *GNAQ* transcript showed specific knockdown of the mutant Gaq while sparing the product of WT *GNAQ* transgene (Figure 2a and b). The same specificity was observed for one out of six siRNAs targeting mutant *GNA11* transcript (Figure 2c and d)

### Inositol-1-phosphate (IP1) assay

Intracellular concentrations of IP1, downstream metabolite of inositol tris-phosphate, which is key mediator of intracellular calcium signal, were quantified in TIME transgenic cells using HTRF-IP-One kit (Cisbio Bioassays) as per the manufacturer's instructions. For IP1 experiments after *GNAQ or GNA11*-mutant silencing, TIME cells were transfected with siRNAs in antibiotic-free complete medium, medium was replaced 18 hours after transfection and the IP1 assay was performed 48 hours after transfection. Briefly, TIME cells were trypsinized, and cell pellets resuspended in complete medium and transferred to a 384-well microtitre plate at a density of 50,000 cells per 7 μl in each well, and a total of 5–6 wells were used as technical replicates for each experimental condition. To each well 7 μl of stimulation buffer were added. After 90 minutes of incubation at 37 °C, 3 μl of IP1-d2 conjugate and 3 μl of europium cryptate–labelled anti-IP1 antibody dissolved in lysis buffer were added to the cells. After incubation in the dark for 1 hour at room temperature, fluorescence was sequentially measured at 620 and 665 nm in every well by Tecan Spark plate reader. To account for potential errors in cell counting, 3 μl from the same cell suspensions used for IP1 assay were also seeded in individual wells of a 96-well plate in quadruplicate for each condition and assayed by CellTiter-Blue Cell Viability Assay (Promega, Madison, WI) shortly after attachment of the cells to the surface of the wells (6 hours). Results from IP1 assay were then further normalised to CellTiter-Blue readings.

### Luciferase assay

HEK DKO Gaq/11;CASR;NFAT-Luc cells were seeded at density of 10,000 cells per well in 96-well plates and transfected with pcDNA3.1 *GNAQ*^*WT*^, *GNAQ*^*R183Q*^, *GNA11*^*WT*^, or *GNA11*^*R183C*^ plasmids (Lipofectamine 2000) using 40 ng, 5 ng, 5 ng, and 4 ng of constructs, respectively, to obtain similar expression levels of cDNAs. The day after transfection, cells were starved for 16 hours in DMEM containing 25 mM HEPES, 0.45 mM CaCl_2_ and 0.01% fetal bovine serum. After starvation, cells were treated with different concentrations of CaCl_2_ in calcium-free DMEM containing 25mM HEPES for 4 hours and then lysates were collected in Passive Lysis Buffer (Promega). Lysates were transferred to 96-well assay plates and Firefly luciferase activity was measured from individual wells by addition of luciferase assay reagent (Promega cat. E1501) using a plate reader with automatic injector (PHERAstar) and following the manufacturer’s instructions.

### Fluo-8 AM assay

Cells were seeded at density of 8000 cells per well in 96-well plates and the next day were incubated in 2 mM Fluo-8 AM-HBSS for 60 minutes at 37 °C, before replacing the dye-containing solution with HBSS and incubating for another 30 minutes at room temperature. Cells were stimulated with thrombin 10× solution in HBSS (final concentration 1U/ml) and fluorescence recorded every second (excitation 490 nM/emission 525 nM) using a plate reader with automatic injector (PHERAstar).

For experiments in HBSS-free buffer, cells were incubated in HBSS calcium-free for 100 seconds before stimulation by thrombin.

For analysis of cells treated with siRNAs, cells were transfected 48 hours before Fluo-8 AM loading.

Cytosolic calcium concentration was calculated using: [Ca^2+^]c = KD(F-Fmin)/(Fmax-F), where KD is the constant of dissociation of Fluo-8 for Ca^2+^ (389 nM), Fmax and Fmin are the maximal and minimal fluorescence values determined after addition of CaCl_2_ (10 mM) and Triton (0.1%) in HBSS or BAPTA (10 mM) and Triton (0.1%) in Ca^2+^-free HBSS, respectively.

### Endothelial tube formation assay

Individual wells of a 96-well plate with black walls were coated with 33 μl per well growth factor–reduced Geltrex (catalog A1413202), or 120 μl per well were used to coat wells in 24-well black plates. TIME *GNAQ* WT or R183Q were trypsinized, counted and seeded in individual wells of a 96-well plate (3,000 cells/well) or in 24-well plate (25,000 cells/well). Thirty minutes before the end of the incubation period of 8 hours, calcein AM (catalog C3099) was added to each well at a final concentration of 2 μg/ml. Tube formation was imaged with a ×4 objective lens of EVOS FLoid Imaging System microscope. The degree of tube formation was assessed by measuring total length of segments, branches, and isolated elements in triplicate using randomly chosen fields from each well using the angiogenesis analyzer for ImageJ (http://image.bio.methods.free.fr/ImageJ/?Angiogenesis-Analyzer-for-ImageJ).

### Statistical analysis

Differences between means were analysed by unpaired or paired *t*-tests, assuming equal variances. Time-signal intensity curves were compared by two-way ANOVA.
